# Design and Optimization of Diclofenac Sodium Controlled Release Solid Dispersions by Response Surface Methodology

**DOI:** 10.4103/0250-474X.40327

**Published:** 2008

**Authors:** H. N. Shivakumar, B. G. Desai, G. Deshmukh

**Affiliations:** Department of Pharmaceutical Technology, K. L. E. S's College of Pharmacy, Rajajinagar 2^nd^ Block, Bangalore - 560 010, India

**Keywords:** Solid dispersions, diclofenac sodium, Eudragit RS 100, Eudragit RL 100, coevaporates

## Abstract

A 3^2^ factorial design was employed to produce controlled release solid dispersions of diclofenac sodium in Eudragit RS and RL by coevaporation of their ethanol solution in a flash evaporator. The effect of critical formulation variables namely total polymer pay loads and levels of Eudragit RL on percent drug incorporation (% DI), drug release at the end of 12 hours (Rel_12_) and drug release at the end of 3 hours (Rel_3_) were analyzed using response surface methodology. The parameters were evaluated using the F test and mathematical models containing only the significant terms were generated for each parameter using multiple linear regression analysis and analysis of variance. Both the formulation variables studied exerted a significant influence (*p* < 0.05) on the drug release whereas the total polymer levels emerged as a lone factor significantly influencing the percent drug incorporation. Numerical optimization technique employing desirability approach was used to develop a new formulation by setting constraints on the dependent and independent variables. The experimental values of % DI, Rel_12_ and Rel_3_ for the optimized batch were found to be 95.22 ± 1.13%, 74.52 ± 3.16% and 29.37 ± 1.26% respectively which were in close agreement with those predicted by the mathematical models. The Fourier transform infrared spectroscopy, Differential scanning calorimetry and Powder x-ray diffractometry confirmed that the drug was reduced to molecular or microcrystalline form in the hydrophobic polymeric matrices, which could be responsible for the controlled drug release from the solid dispersions. The drug release from the solid dispersions followed first order rate kinetics and was characterized by Higuchian diffusion model.

The solid dispersion technique was originally utilized to enhance the dissolution of poorly water soluble drugs using water soluble inert carriers^1^,^2^. However the same technique has been employed for preparing sustained release forms of soluble drugs using water insoluble carriers^3^,^4^. Solid dispersions prepared using water insoluble carriers have been aimed at optimizing pharmacokinetics and reduce gastrointestinal side effects of non steroidal anti-inflammatory drugs^5^,^6^. Eudragit RS and RL are methacrylic acid copolymers containing quaternary ammonium groups. The incorporation and the release of non steroidal antiinflammatory drugs (NSAIDs) from RS and RL polymers have been shown to be strongly dependent on the acidic nature and pK_a_ values of these drugs, which allows physical interactions to occur with the ammonium group on the RS and RL back bone^7^. Literature citations reveal that solid dispersions of NSAIDs like diflunisal^8^ and flubriprofen^9^ have been developed of late using Eudragit RS and RL with the aim to prolong the drug release and minimize the gastric side effects.

Diclofenac, a phenyl acetic acid derivative is a NSAID with a potent cyclooxygenase inhibiting action. The drug is prescribed for long term treatment of rheumatoid arthritis, osteoarthritis and ankylosing spondylitis. Gastrointestinal side effects such as bleeding, ulceration or perforation of the intestinal wall are commonly seen when the drug is administered orally^10^. Diclofenac when administered orally is absorbed well and undergoes extensive first pass metabolism resulting a terminal half life of 1 to 2 hours which requires frequent administration of 50 mg thrice a day^11^. The present work was aimed to modulate the drug release from the solid dispersions using a combination of two insoluble, pH insensitive acrylic polymers namely Eudragit RS and Eudragit RL. The first part of the work was aimed to systematically analyze the influence of the two polymers on the drug release using a 3^2^ factorial design. The second part of the study involved development of an optimized solid dispersion formulation with desirable drug incorporation and release features by numerical optimization technique. The multiparticulate systems has been proposed as they are known to spread uniformly through out more uniformly in the gastro-intestinal tract and reduce the local irritation as compared the single-unit dosage forms^12^. Furthermore administration of NSAIDs incorporated multiparticulates can avoid undesired intestinal retention of polymeric material, which can occur in case of a single unit form, particularly on chronic dosing^12^.

## MATERIALS AND METHODS

Diclofenac sodium was a gift sample from Zydus Health Care Ltd., Bangalore. Eudragit RS-100 and RL-100 manufactured by Rohm Pharma, Darmstadt, Germany were generously donated by Degussa, Mumbai. Potassium dihydrogen orthophosphate, disodium hydrogen orthophosphate and sodium hydroxide were purchased from S. D. Fine Chemicals, Mumbai. All other chemicals and regents used were of AR grade.

### Experimental Design:

A 2 factor 3 levels full factorial design was employed to design controlled release solid dispersions of diclofenac sodium^13^. This design was suitable for exploring quadratic response surfaces and constructing second order polynomial models. The two independent formulation variables analyzed during the study were total polymer payloads in the solid dispersion (X_1_) and the proportion of Eudragit RL 100 in the total polymer (X_2_). The selected factors with the actual and coded levels as per the design are represented in Table 1. The higher, lower and the intermediate levels of each factor are coded as +1, −1 and 0, respectively. The dependent variables investigated were percentage drug incorporation (Y_1_) and drug release at the end of 12 h (Y_2_). In order to have an insight on the burst effect in pH 7.4, drug released at the end of 3 h (Y_3_) was studied as the third response parameter.

**TABLE 1:** FACTOR COMBINATIONS AND RESPONSE PARAMETERS OF SOLID DISPERSIONS OF DICLOFENAC SODIUM PREPARED AS PER 3^2^ FACTORIAL DESIGN

Batch code	X_1_*	X_2_*	% DI (%w/w)^a^	Rel_12_ (%)^a^	Rel_3_ (%)^a^
F1	−1 (50)	−1 (0)	86.75 ± 1.12	76.74 ± 4.23	29.83 ± 1.54
F2	0 (65)	−1 (0)	89.62 ± 0.50	70.44 ± 3.56	21.96 ± 1.75
F3	+1 (80)	−1 (0)	95.10 ± 0.62	52.12 ± 2.45	15.11 ± 1.23
F4	−1 (50)	0 (20)	85.47 ± 1.48	85.22 ± 3.68	34.25 ± 1.58
F5	0 (65)	0 (20)	90.02 ± 0.91	79.56 ± 4.02	28.22 ± 1.56
F6	+1 (80)	0 (20)	94.73 ± 1.21	65.41 ± 3.25	24.56 ± 1.23
F7	−1 (50)	+1 (40)	84.20 ± 1.61	96.44 ± 1.38	42.56 ± 1.89
F8	0 (65)	+1 (40)	91.30 ± 0.69	88.79 ± 4.13	36.78 ± 1.66
F9	+1 (80)	+1(40)	95.38 ± 1.15	78.12 ± 3.86	32.56 ± 1.42

The parentheses in the data represent the decoded factor levels. X_1_ represents polymer payloads; X_2_ represents % w/w of Eudragit RL in the total polymer. % DI = Percentage drug incorporation; Rel_12_ = Release at the end of 12 h; Rel_3_ = Release at the end of 3 h.

a The values represent the average of three determinations (*n* = 3)

### Formulation of Eudragit solid dispersions containing diclofenac sodium:

Eudragit solid dispersions containing diclofenac sodium were prepared by solvent method^9^. The polymer mixture consisting of Eudragit RS and RL was accurately weighed and dissolved in absolute ethanol to get a clear solution (5%w/w). Accurately weighed amount of drug at different drug to polymer ratios was dissolved in the polymer solution and stirred on a magnetic stirrer (model 2 MLH, Remi motors, Mumbai) at room temperature for duration of 4 to 6 h. The solvent was removed under reduced pressure (100 mm Hg) in a rotary flash evaporator (Buchi type, Servewell instruments Inc., Bangalore) at an external maximum temperature of 40°. The solid residue obtained was dried in a vacuum oven (Lab model, Servewell instruments Inc., Bangalore) at 30° for 24 h. The powder was passed through #44 mesh (355 μm) and stored in closed glass container. Nine batches of solid dispersions (F1 to F9) were prepared as per 3^2^ factorial design by varying the polymer loads, percent of Eudragit RL 100 in the total polymer and maintaining the other experimental conditions constant.

### Drug content estimation:

The drug content of the solid dispersion was determined in triplicate by equilibrating an accurately weighed quantity of the solid dispersion in buffer of pH 7.4 for a period of 24 h. The samples were filtered, suitably diluted and assayed spectrophotometrically at 276 nm in Jasco V-530 UV/Vis spectrophotometer. The polymers did not interfere with the drug extraction and assay at the given wavelength.

### *In vitro* drug release studies:

The *in vitro* dissolution studies of the solid dispersions were performed for a period of 12 h in USP XXIII basket dissolution apparatus (TDT-08 T, Electrolab (I) Ltd., Mumbai) following rotating basket method^14^. The studies were carried out initially in 900 ml of 0.1 N HCl (pH 1.2) for the first two hours followed by phosphate buffer of pH 7.4 for the next 10 h. Solid dispersions containing 100 mg of the drug were loaded in the basket of the apparatus and rotated at stirring speed of 100 rpm in the dissolution media maintained at 37±0.5°. Aliquots of samples were withdrawn every hour, filtered through 0.45 μm filter, diluted suitably, and assayed spectrophotometrically at 273 nm for samples with pH 1.2 and at 276 nm for samples with pH 7.4. The raw dissolution data recorded in triplicate was analyzed to calculate the amount of drug released and percentage cumulative drug released at different time intervals. The dissolution data in pH 7.4 was treated using different mathematical equations to characterize the mechanism and kinetics of drug release.

### Regression analysis:

The targeted response parameters were statistically analyzed by applying one-way ANOVA at 0.05 level in Design-Expert® 6.0.5 demo version soft ware (Stat-Ease Inc., Minneapolis, Minnesota)^15^. The individual parameters were evaluated using the F test and quadratic models of the form Y = β_0_ + β_1_ X_1_ + β_2_ X_2_ + β_3_ X_1_ X_2_ + β_4_ X_1_^2^ + β_5_ X_2_^2^ were generated for each response parameter using multiple linear regression analysis (MLRA), where Y is the level of the measured response; β_0_ is the intercept β_1_ to β_5_ are the regression coefficients. X_1_ and X_2_ stand for the main effects; X_1_X_2_ is the interaction between the main effects; X_1_^2^ and X_2_^2^ are the quadratic terms of the independent variables that were used to simulate the curvature of the designed sample space. A backward elimination procedure was adopted to fit the data into different predictor equations. The quadratic models generated by regression analysis were used to construct the 3-dimensional graphs in which response parameter Y was represented by a curvature surface as a function of X. The effect of the independent variables on each response parameters was visualized from the contour plots.

Numerical optimization using desirability approach was employed to locate the optimal settings of the formulation variables to obtain the desired response^16^. An optimized formulation was developed by setting constraints on the dependent and independent variables. The new formulation was evaluated for the responses and the experimental values obtained were compared with those predicted by the mathematical models.

### Scanning electron microscopy:

Scanning electron microscopy has been employed to study the morphology and surface topography of the solid dispersions^16^. The solid dispersions from the optimized batch were mounted on the SEM sample stab, using a double-sided sticking tape and coated with gold (200 A°) under reduced pressure (0.001 torr) for 5 min using an Ion sputtering device (Jeol JFC-1100 E, Tokyo, Japan). The gold coated samples were observed under the scanning electron microscopy (SEM-Jeol JSM-840 A, Tokyo, Japan) and photomicrographs of suitable magnifications obtained.

### Fourier transform infrared spectroscopy:

The IR spectra of the pure drug, polymer mixture and optimized solid dispersion formulation were obtained to prove the chemical integrity of the drug in the solid dispersions^8^. The samples were powdered and intimately mixed with dry powdered potassium bromide. The mixtures got were taken in a diffuse reflectance sampler and IR spectra recorded by scanning in the wavelength region of 400 to 4000 cm^−1^ in a FTIR Spectrophotometer (model 460 Plus, Jasco, Japan).

### Thermal analysis:

Differential scanning calorimetry (DSC) has been one of the widely employed calorimetric methods to study the solid state of the drug in solid dispersions^8^. Samples of the drug, polymer mixture and the optimized solid dispersion formulation were taken in a flat round bottomed aluminum pans and heated in a temperature range of 50 to 300° at a rate of 10° per minute with purging of nitrogen (50 ml/min) using alumina as reference standard in a differential scanning calorimeter (pyris-1, Perkin Elmer, Boston, USA).

### Powder x-ray diffraction studies:

Powder x-ray diffraction analysis has been used along with DSC to characterize the physical state of the drug in the polymeric matrices of solid dispersions^8^. The diffraction studies of the drug, polymer mixture and the optimized solid dispersion formulation were performed in a powder x-ray diffractometer with a vertical goniometer (PW 1050/37, Philips, Netherlands). PXRD patterns were recorded using monochromatic Cu K α radiation with Ni filter at a voltage of 40 kV and a current of 20 mA between 5 to 80° 2θ values.

## RESULTS AND DISCUSSIONS

Solvent method has been employed recently to produce solid dispersions of NSAIDs in Eudragit RS/RL^8^,^9^. The present investigation was aimed to prepare solid dispersions of diclofenac sodium by solvent method as per 3^2^ factorial design utilizing two insoluble acrylic polymers RS and RL in combination. Eudragit RS has a lower a lower content of quaternary ammonium groups (4.5 to 6.8%), and is considered less permeable to water as compared to the more permeable RL (8.8 to 12% ammonium groups)^17^. They are insoluble at the physiological pH values and are capable of swelling. These polymers are reported to display strong electrostatic interaction with acidic drugs having a carboxylic moiety and low pK_a_ values, which influenced the drug release^9^. Many researchers have utilized a combination of these two polymers to modulate the drug release from solid dispersions^16^,^18^. Absolute alcohol was employed as a common solvent to dissolve the polymers as well as the drug.

A set of preliminary trials were undertaken to establish the range of the each formulation variable with the aim to optimize the drug release from the solid dispersions. The preliminary trials conducted revealed that solid dispersions with low polymer loads (<50%) resulted in quick drug release (T_50_ < 3 h) whereas those prepared with high polymer loads (>80%) were rubbery, scarcely manageable and resulted in poor drug release (T_50_ > 12 h). Based on these observations the lower and higher levels of polymers were retained at 50% and 80%, respectively during the run.

As drug release from solid dispersion produced with Eudragit RS 100 alone was inherently sustained, Eudragit RL 100 by virtue of its higher permeability was used to enhance the drug release from the solid dispersions. Since solid dispersions prepared with higher amounts of Eudragit RL 100 (>40%) resulted in quicker drug release (T_50_ < 3 h), the levels of Eudragit RL 100 were varied between 0% and 40% of the total polymer loads respectively during the study. The other formulation variables such as the batch size, concentration of the polymer in absolute alcohol and the processing variables like external pressure (100 mm of Hg) and temperature (40°) during evaporation were maintained constant during the study.

**Fig. 1:**
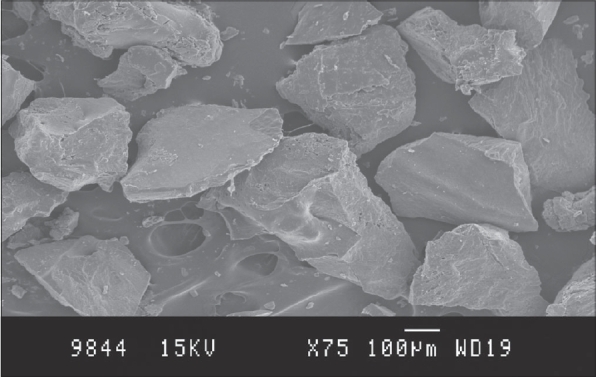
SEM Photomicrograph. Photomicrographs of diclofenac sodium loaded solid dispersions as revealed by scanning electron microscopy.

Scanning photomicrographs of the solid dispersions from the optimized batch are displayed in fig. 1. It was evident from the photomicrographs that the drug was dispersed in crystalline form in the Eudragit polymeric matrix. The polymeric matrix would have been saturated with the drug and the excess drug may be crystallized as irregular flakes.

**Fig. 2:**
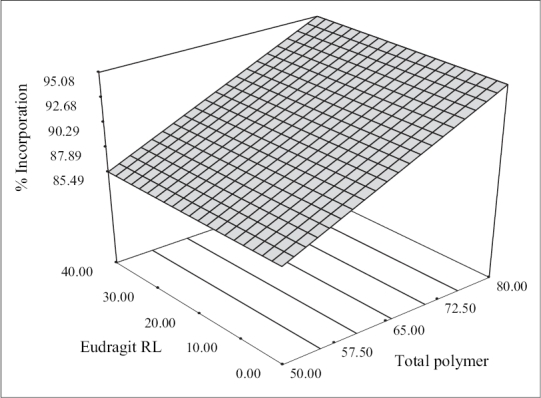
Response surface plot showing the effect of formulation variables on percent drug incorporation. 3-D surface plots showing the effect of total polymer pay loads and Eudragit RL on percent drug incorporation.

The percentage drug incorporation for different batches of solid dispersions produced is displayed in Table 1. The percent incorporation values depended on the total polymer payloads and were found to range from 84.20±1.61 to 95.38±1.15. The linear model generated for percent drug incorporation (Y_1_ = 90.29+4.80 X_1_) was found to be significant with an F value of 193.31 (*p* < 0.0001) and R^2^ value of 0.965. The model indicated that the polymer payload emerged as the lone factor, which exerted a significant influence on the percent incorporation. The 3-D plot (fig. 2) shows that the percent incorporation increased from 86.75±1.12 to 95.10±0.62 and from 84.20±1.61 to 95.38±1.15 at lower and higher levels of Eudragit RL respectively as the total polymer levels increased. The improvement in percent drug incorporation with increased polymer levels was due to the fact that more drug gets dissolved and incorporated in the polymer matrix with increase in the polymer payloads. A linear relationship between the two variables investigated on the percent drug incorporation was clearly seen from the corresponding contour plots which suggested that percent drug incorporation can be enhanced using high polymer pay loads.

The amount of drug released from the solid dispersions at the end of 12 h of dissolution is shown in Table 1. A poor drug release ranging from 1.60±0.38 to 3.14±0.58 was observed from the solid dispersions in pH 1.2. This can be attributed to the pH dependent solubility of the drug, which is reported to increase at pH values higher than the pK_a_ (4.0) of the drug^19^. The drug release from the solid dispersions in pH 7.4 depended on the total polymer payloads as well as the Eudragit RL levels. Solid dispersions produced with Eudragit RS alone showed slow drug release ranging from 52.12±2.45% to 76.74±4.23% by the end of 12 h of dissolution. The slow drug release from solid dispersions with Eudragit RS can be attributed to the low permeability of the polymer, which posed a significant hindrance to fluid penetration and passive drug diffusion.

Incorporation of the Eudragit RL is reported to enhance the drug release from the solid dispersions^7^. The drug release from solid dispersions in which 20% of the Eudragit RS is replaced with Eudragit RL was found to range from 65.41±3.25 to 85.22±3.68% after 12 h of dissolution signifying an enhancement of drug release in presence Eudragit RL. A burst effect accounting for drug release ranging from 24.56±1.23 to 34.25±1.58 was observed once these dispersions were exposed to pH 7.4.

Solid dispersions in which 40% of Eudragit RS was substituted with Eudragit RL further enhanced the drug release as 78.12±3.86 to 96.44±1.38 of the drug was released at the end of the dissolution period. A burst release ranging from 32.56±1.42 to 42.56±1.89 was observed from these formulations once they were exposed to alkaline pH.

**Fig. 3:**
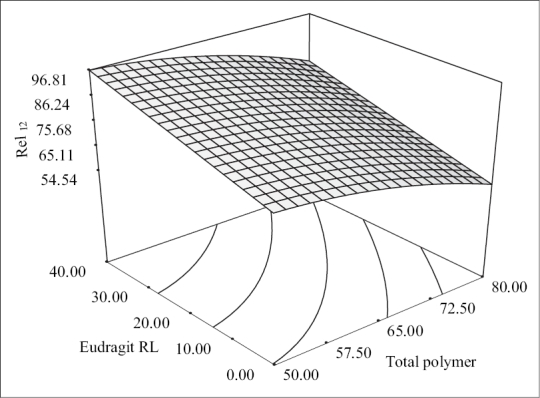
Response surface plot showing the effect of formulation variables on percent release at the end of 12 h. 3-D surface plot showing the effect of total polymer pay loads and Eudragit RL on percent release at the end of 12 h.

The second order polynomial model (Y_2_ = 79.60 − 1046X_1_ + 10.67X_2_ − 3.92X_1_^2^) generated for release at the end of 12 h was significant with F value of 127.05 (*p* < 0.0001) and R^2^ value of 0.987. The quadratic model generated revealed that the total polymer payloads and levels of Eudragit RL had a significant antagonistic influence on the drug release without producing any interaction. The response surface plots (fig. 3) illustrate that the drug release at the end of 12h decreased from 76.74±4.23% to 52.12±2.45% and from 96.44±1.38 to 78.12±3.86% at low and high levels of Eudragit RL respectively as the polymer loads increased. This can be ascribed to increased amounts of drug getting solubilized in the polymeric matrix at higher polymer levels leaving fewer drug crystals on the surface for dissolution. The quicker drug release observed at low polymer loads can be attributed to increased drug crystals positioned at the surface, which got exposed and dissolved in the dissolution fluid.

Eudragit RL levels were found to have a positive influence on the drug release as the release was enhanced on incorporation of Eudragit RL. It was evident from the 3-D plots that the drug release at the end of 12 h increased from 76.74±4.23% to 96.44±1.38% and from 52.12±2.45% to 78.12±3.86% at low and high polymer levels respectively as the Eudragit RL levels increased. This enhancement in drug release can be due to increased permeability of Eudragit RL because of the greater quaternary ammonium group content in the polymer. A curvilinear relationship between the two independent variables on Rel_12_ was evident from the corresponding contour plots, which indicated that Rel_12_ could be maximized using high levels of Eudragit RL at low polymer payloads.

**Fig. 4:**
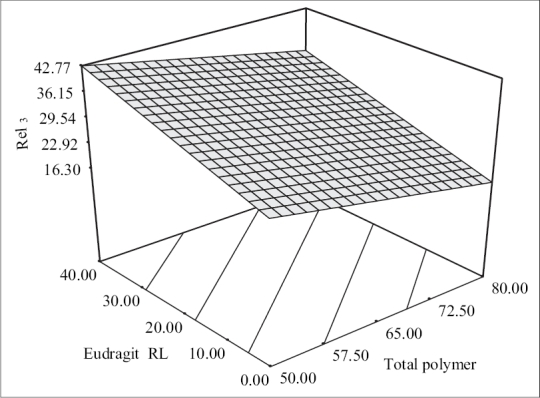
Response surface plot showing the effect of formulation variables on percent release at the end of 3 h. 3-D surface plot showing the effect of total polymer pay loads and Eudragit RL on percent release at the end of 3 h.

The predictor equation (Y_3_ = 29.54 − 5.74 X_1_ + 7.50 X_2_) generated for release at the end of 3 h was significant with F value of 148.83 (*p* < 0.0001) and R^2^ value of 0.980. The polymer loads were found to have a negative influence on the burst release whereas Eudragit RL levels had a positive influence on the burst release. The response surface plots (fig. 4) illustrate that the burst release at the end of third hour decreased from 29.83±1.54% to 15.11±1.23% and from 42.56±1.89 to 32.56±1.42% respectively at low and high levels of Eudragit RL, respectively as the polymer loads increased. This can be due to better incorporation of the drug in the polymeric matrices, which left behind fewer drug crystals on the surface with increase in the polymer loads.

The levels of Eudragit RL levels were found to have a positive influence on the burst release. It was evident from the 3-D plots that the drug release at the end of three hours increased from 29.83±1.54% to 42.56±1.89% and from 15.11±1.23% to 32.56±1.42% at low and high polymer levels respectively as the Eudragit RL levels increased. This increase in burst effect was due to increased permeability and hydrophilicity of Eudragit RL because of the higher content of quaternary ammonium group in the polymer. A linear relationship between the two independent variables on Rel_3_ was clear from the corresponding contour plots, which also indicated that burst drug release could be minimized using low levels of Eudragit RL at high polymer levels.

The mechanism of drug release from the solid dispersions was found to be diffusion controlled as plots of percent cumulative drug release versus square root of time was found to be linear with the regression coefficient (R^2^) values ranging from 0.975±0.04 to 0.999±0.003 for the nine formulations. The values of the Higuchi rate constant was found to vary between 17.38±0.52 to 25.22±0.72% h^−½^ showing an increasing trend with decrease in the polymer payloads and increase in Eudragit RL levels. The kinetics of drug release from the solid dispersions was found to follow first order kinetics as the plots of log concentration of drug retained versus time were linear with the values for first order rate constants ranging from 0.060±0.002 to 0.29±0.01h^−1^.

A numerical optimization technique using the desirability approach was employed to develop a new formulation with the desired responses. Constraints like maximizing the percentage drug incorporation (X_1_) and release at the end of 12 hours (X_2_) in addition to minimizing the burst release (X_3_) were set as goals to locate the optimum settings of the independent variables in the new formulation. The optimized solid dispersion formulation was developed in taking 21.46% of diclofenac sodium, 39.44% of Eudragit RS and 23.83% of Eudragit RL. The optimized formulation was evaluated for percentage drug incorporation and drug release. Table 2 enlists the value of the observed responses and those predicted by mathematical models long with the percentage prediction errors. The prediction error for the response parameters ranged between 1.50 and 3.15 with the value of absolute error of 2.36±0.70. The low values of error establish the ability of response surface methodology to predict the behavior of the drug loaded solid dispersions. Model reduction using MLRA by eliminating the insignificant terms with *p* > 0.05 has already been reported to result in better prognosis of the performance of the optimized formulation^20^. The drug release from the optimized formulation was found to follow first order kinetics and was characterized by Higuchi diffusion model.

**TABLE 2:** COMPARISON OF THE EXPERIMENTALLY OBSERVED RESPONSES OF THE OPTIMIZED SOLID DISPERSION FORMULATION WITH THAT OF THE PREDICTED RESPONSES

Response parameters	Constraints set	Observed values*	Predicted values	% Error
DI (%)	Minimize	95.22 ± 1.13	94.62	0.60
Rel_12_ (%)	Maximize	74.52 ± 3.16	72.50	2.02
Rel_3_ (%)	Minimize	29.37 ± 1.26	28.26	1.11

* The values represent the average of three determinations (*n* = 3)

**Fig. 5:**
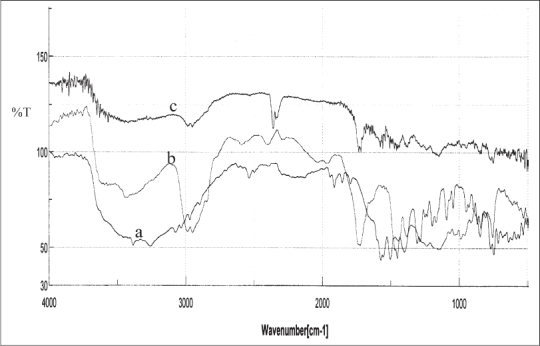
IR Spectra. IR spectra of diclofenac sodium (a), Eudragit mixture (b) and drug loaded solid dispersions.

The IR spectra of the diclofenac sodium, polymer mixture and drug loaded solid dispersions ware portrayed in fig. 5. The IR spectra of diclofenac sodium exhibited distinctive peaks at 3381.57 cm^−1^ due to NH stretching of the secondary amine, 1572.66 cm^−1^ owing to –C = O stretching of the carboxyl ion and at 745.35 cm^−1^ because of C-Cl stretching. The FTIR spectra of Eudragits displayed characteristic peaks at 2981.41 due to CH aliphatic stretching and at 1724.05 due to –C = O stretching. In the IR spectra of the solid dispersions the peak due to the drug carboxyl group was shifted to 1577.49 cm^−1^ whereas the signal resulting from the polymer carboxyl appeared at 1734.66 cm^−1^ ruling out the possibility of any chemical interaction between the drug and Eudragit polymers. A weak electrostatic interaction between the carboxyl group of the drug and the ammonium group of the polymers during co-dissolution and solvent evaporation could not be ruled out. A weak electrostatic interaction between the carboxyl group of the NSAIDs and the ammonium group of the polymer has been reported in the literature^9^.

**Fig. 6:**
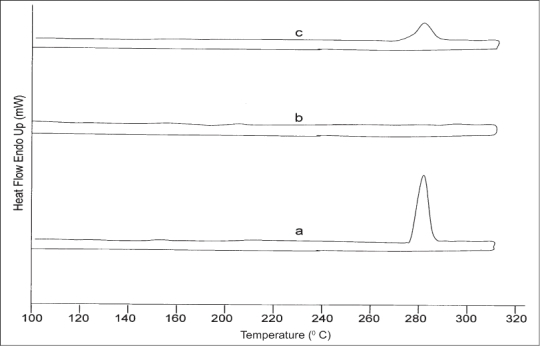
Differential scanning calorimetric scan. DSC thermogram of diclofenac sodium (a), Eudragit mixture (b) and drug loaded solid dispersions.

The DSC thermogram of the drug (fig. 6) depicts a sharp endothermic peak at 280° corresponding to the melting transition temperature of diclofenac sodium^19^. The polymer mixture did not present any thermal transition under the experimental conditions. The drug endothermic peak was suppressed in the thermogram of the solid dispersion suggesting that the drug was able to dissolve partially in the polymer to form a solid-solid solution. The appearance of low intensity endothermic peak also indicated the some of the drug still managed to crystallize out from the saturated Eudragit matrices during coevaporation.

**Fig. 7:**
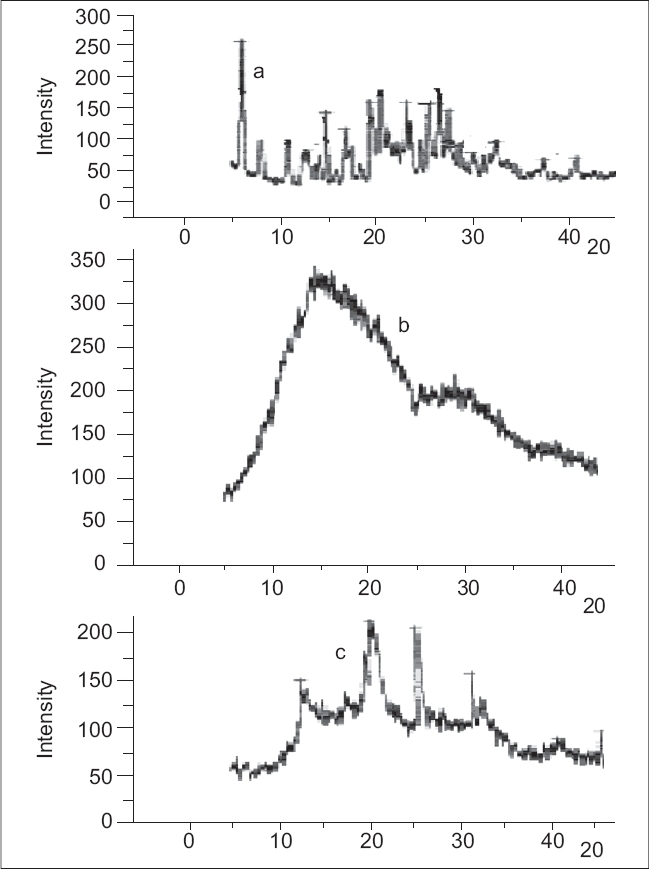
Powder x-ray diffractograms. Powder x-ray diffraction patterns of diclofenac sodium (a), Eudragit mixture (b) and drug loaded solid dispersion.

The PXRD spectra of the diclofenac sodium, polymer mixture and drug loaded solid dispersions ware portrayed in fig. 7.. The amorphous nature of the Eudragit RS and RL was confirmed from the x-ray diffraction analysis. The crystalline nature of the drug was clearly demonstrated by the characteristic PXRD pattern with peaks appearing at 6.38, 8.26, 14.90, 19.23, 20.74, 25.96, 26.82 and 27.70 2θ values. Signals at 13.04, 20.96, 25.96 and 31.70 2θ values attributable to the crystalline drug appeared in the x-ray diffractogram of the solid dispersion confirmed the existence of the drug probably in the microcrystalline form in the polymer matrix. Microcrystallization of NSAIDs from the Eudragit matrices when the drug concentration exceeded its solubility in the polymer matrix has been cited in literature^8^,^9^. The DSC and PXRD studies collectively indicated that a portion of the drug incorporated existed as solid-solid solution in the polymer matrix whereas the excess drug crystallized in the microcrystalline form from the polymeric matrices. The presence of the drug as a solid-solid solution in the hydrophobic polymeric matrix could be responsible for the controlled drug release from the solid dispersions. Solid solutions in hydrophobic polymers are responsible to prolong the drug release from multiparticulate systems^21^.

A 3^2^ factorial design has been employed to produce solid dispersions of diclofenac sodium in Eudragit polymers by solvent method. Both the formulation variables studied exerted a significant influence on the drug release whereas the total polymer had a significant influence on the percent drug incorporation. An optimized formulation with desirable release properties was developed employing numerical optimization technique. The results obtained indicate that response surface methodology can be employed successfully to quantify the effect of several formulation and processing variables thereby minimizing the number of experimental trials and cutting down the formulation development cost.
